# 3D Multi-Spectrum Sensor System with Face Recognition

**DOI:** 10.3390/s131012804

**Published:** 2013-09-25

**Authors:** Joongrock Kim, Sunjin Yu, Ig-Jae Kim, Sangyoun Lee

**Affiliations:** 1 Department of Electrical and Electronic Engineering, Yonsei University, 134 Shinchon-dong, Seodaemun-gu, Seoul 120-749, Korea; E-Mail: jurock@yonsei.ac.kr; 2 Department of Broadcasting and Film, Cheju Halla University, 38, Halladaehak-ro, Jeju-si, Jeju-do 690-708, Korea; E-Mail: sjyu@chu.ac.kr; 3 Imaging Media Research Center, Korea Institute of Science and Technology, Hwarang-ro 14-gil 5, Seongbuk-gu, Seoul 136-791, Korea; E-Mail: kij@imrc.kist.re.kr

**Keywords:** image sensor, depth sensor, sensor fusion, face recognition

## Abstract

This paper presents a novel three-dimensional (3D) multi-spectrum sensor system, which combines a 3D depth sensor and multiple optical sensors for different wavelengths. Various image sensors, such as visible, infrared (IR) and 3D sensors, have been introduced into the commercial market. Since each sensor has its own advantages under various environmental conditions, the performance of an application depends highly on selecting the correct sensor or combination of sensors. In this paper, a sensor system, which we will refer to as a 3D multi-spectrum sensor system, which comprises three types of sensors, visible, thermal-IR and time-of-flight (ToF), is proposed. Since the proposed system integrates information from each sensor into one calibrated framework, the optimal sensor combination for an application can be easily selected, taking into account all combinations of sensors information. To demonstrate the effectiveness of the proposed system, a face recognition system with light and pose variation is designed. With the proposed sensor system, the optimal sensor combination, which provides new effectively fused features for a face recognition system, is obtained.

## 1. Introduction

Progress in computer vision and sensor technology has made it possible to acquire various types of information, such as two-dimensional (2D) data at different wavelengths, as well as three-dimensional (3D) information in real-time [1–4]. Since each type of sensor provides different features under various environmental conditions, the performance of an application depends highly on selecting a sensor or multi-sensor combination. Therefore, the selection of appropriate sensors has become one of the most significant factors for high-performance vision systems [2,5,6].

Most of the early computer vision applications were based on visible (RGB) sensors, since a visible image represents what humans perceive visually [1,6]. The range of wavelengths correspond to a frequency range from approximately 380 nm to 700 nm. Images also contain texture information, which is one of the most important types of information for object detection, tracking and classification [7,8]. However, since visible sensor images are strongly distorted by changes in illumination, the performance of visible-sensor-based systems also depends highly on illumination conditions.

The wavelength range of near-infrared (IR) and thermal-IR sensors are from 0.7 μm to 1.0 μm and from 9 μm to 14 μm, respectively. Although these wavelengths are not perceived by the human visual system, IR images contain distinctive features, such as thermal radiation emitted by objects. Furthermore, compared to visible sensor images, infrared images are more invariant to visible illumination changes [9,10]. However, neither near-IR nor thermal-IR images include color (RGB) information. In addition, thermal-IR images do not contain texture information and can be affected by ambient temperature.

Recently, real-time 3D depth sensors, such as the Kinect and time-of-flight (ToF) sensors, have been introduced and have become some of the most useful sensors for vision applications [3,4]. Real-time 3D sensors make it possible to analyze detailed 3D shape information that cannot be acquired by 2D sensors. Since the pixel value of a depth image represents the distance between a camera and an object, many researchers have attempted to apply these sensors to 3D-based applications, such as 3D-based recognition and 3D object modeling. However the distance information from such sensors is highly noisy and cannot support texture information.

As explained above, each sensor provides different information and has advantages and disadvantages in different environments. Therefore, to design more flexible and robust systems, the selection of sensors for a specific application is a very important problem. However, obtaining the optimal sensor combination based on calibrated and fused information from all sensors is very difficult, because of the heterogeneous characteristics of sensors.

In this paper, we propose a sensor system, which we refer to as a 3D multi-spectrum sensor system, consisting of three types of sensors: visible, thermal-IR and ToF sensors. Through the registration of all sensors, visible, near-IR, thermal-IR and 3D information is integrated into a 3D multi-spectrum data framework in real-time. In the data framework, all information from all sensors is calibrated and can be easily fused. With the data framework, we can easily select optimal sensor combinations considering all combinations of sensor information. To show the effectiveness of the proposed system, we apply the system to face recognition in the presence of light and pose variations. With the proposed system, we can obtain fused optimal features for high-performance face recognition. In addition, the proposed system can also be used for surveillance, 3D object modeling and object and human recognition.

This paper is organized as follows. In the next section, we briefly review the related state-of-the art research areas. The proposed sensor system and its application with recognition methods are discussed in Section 3. Section 4 shows experimental results on the use of 3D multi-spectrum face data in face recognition. Conclusions are given in Section 5.

## 2. Related Works

Previous works related to this paper are roughly divided into three research areas: sensor registration, applications of time-of-flight cameras and face recognition. Since the proposed system consists of three different sensors (visible, thermal-IR and ToF sensors) at different locations, the images from the sensors need to be registered. Therefore, sensor registration is introduced in this chapter. In addition, recent studies using ToF camera and face recognition in terms of light and pose variation are presented as applications of the proposed system.

### 2.1. Sensors Fusion

Various sensors, such as visible, thermal-IR and 3D sensors, have been introduced into the commercial market. Since each sensor provides different features, many approaches for combining features to improve the performance of the system have been proposed. In order to fuse sensor data, registration to transform different sets of data into one coordinate system should be accomplished.

Many approaches have been proposed for registering different types of images, such as visible, IR and 3D. In [11], a simple registration between IR and visible images using SIFTis proposed. In [12,13], registration between visible and thermal-IR image data for face recognition involving illumination variations is described. A real-time fusion method of multiple passive imaging sensors, visible, IR, and 3D LADARimaging, is presented in [14]. In [15], a method to register a pair of images captured from visible and IR sensors by line and point matching is presented. In addition, calibration between depth and color sensors by a maximum likelihood solution by using checker board is performed in [16,17]. In [18], a multiple sensor fusion system, which combines RGB-Dvision, lasers and a thermal sensor, in order to detect people, in a mobile robot.

Even though there have been many attempts to develop registration between different types of 2D sensors, perfect registration cannot be achieved using only 2D information. Additionally, even though many approaches for calibration between color and depth images have been proposed, high-performance registration between IR and 3D is still a challenging problem. Moreover, there have thus far been no attempts to register three types of sensors: IR, visible and 3D sensors.

### 2.2. Applications of Time-of-Flight Camera

Recently, a ToF camera, which generates full-range distance data in real-time, has been used to extend the application range of 3D data to real-time systems, such as human-computer interaction (HCI) [19,20], surveillance [21,22] and robotics [23–26]. One of the most useful applications of the ToF camera is 3D object modeling in order to minimize errors in 3D data, since this data contains noise, due to the motion and orientation of the object to be acquired, as well as the reflectivity of surfaces [27–29]. In [27], 3D object reconstruction exploits sequential distance images captured at different positions. In [29], a 3D shape scanning method with a ToF camera is presented to improve the quality of 3D scans based on filtering and scan alignment techniques. In [28], a method to generate spatially consistent 3D object models by registering 3D data from multiple views is described. In addition, the lack of information from ToF cameras can be compensated for by using additional sensors [30–32]. A real-time segmentation and tracking technique that fuses depth and RGB color data proposed in [30] solves some of the problems in RGB image-based segmentation and tracking, such as occlusions and fast motion. In [31], a real-time 3D hand gesture method using calibration ToF and RGB cameras improves the detection rate, as well as the handling of hand overlap with the face to allow for complex 3D gestures. In [32], both a ToF camera and stereo vision are used to make more accurate depth images, and a generated depth map for augmented reality scenarios is applied.

### 2.3. Face Recognition

Many approaches for face recognition to handle pose and light variation have attempted to use various source data, such as 2D, 3D, thermal-IR and near-IR face data, for computer vision and pattern recognition. Face recognition can be roughly classified into two categories: 2D and 3D data-based face recognition.

In 2D-based face recognition, features invariant under visible light changes are extracted from 2D visible, thermal-IR and near-IR face images. In [33], the techniques for decomposing 2D visible face images into non-negative factors to address illumination changes are presented. In [34], a local ternary pattern (LTP) is proposed that can compensate for the main weaknesses of LBP, including sensitivity to large variations in illumination and to random and quantization noise in uniform and near-uniform image regions. A novel solution for illumination invariant face recognition using near-infrared images and LBP features is proposed in [9]. In [35], a comprehensive and timely review of the literature on the use of infrared imaging for face recognition is presented. In [36], the active appearance model (AAM) is applied to normalize pose and facial expression changes on thermal-IR images, and anatomical features invariant to the exact pattern of facial temperature emissions are extracted for face recognition. In [10], image fusion between visible, near-IR and thermal-IR images is presented, which can enhance the performance of face recognition under uncontrolled illumination conditions. Although IR-image-based face recognition is an effective approach for eliminating visible light changes, the performance still depends on the pose of the face.

One way of dealing with pose and light variations is to use 3D face data, since any face pose can be generated by simple transformations, such as translation, rotation and scaling of 3D face models. In addition, 3D face shape information, such as curvature [37,38], profile [39,40] and range image [41,42], can be extracted from 3D face models, since those features are invariant to pose and light variations. A face recognition system using a combination of color and depth images is proposed in [43,44]. In [45], a novel thermal 3D modeling system using 3D shape, visible and thermal infrared information is proposed that addresses the head pose variation problem in face recognition systems. However, the system cannot acquire thermal 3D data in real-time.

## 3. Proposed 3D Multi-Spectrum Sensor System

In order to integrate various sensor information into one calibrated datum, we propose a novel 3D multi-spectrum sensor system that can provide 3D, visible, near-IR and thermal-IR information. The system consists of ToF, color and thermal-IR sensors. Through the registration step between sensors, we generate calibrated 3D multi-spectrum data in real-time. As an application using the 3D multi-spectrum data, we apply it to a face-recognition system that can address variations in light and pose, as these are the most significant factors causing performance decline [46].

### 3.1. Proposed System

**Figure 1.**
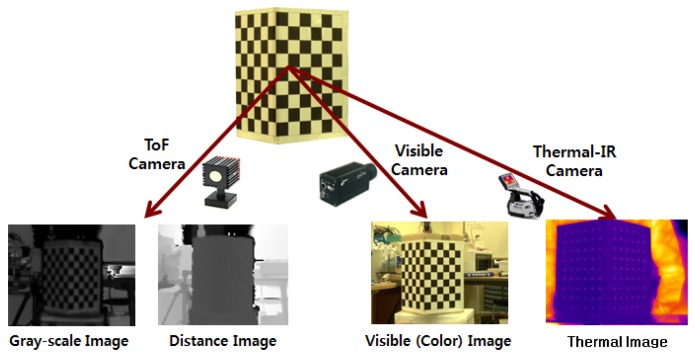
Proposed system comprising ToF, visible and thermal-IR cameras.

Our proposed system consists of ToF, color and thermal-IR cameras, as shown in Figure 1. Although the ToF camera provides depth information and a near-IR (gray-scale) image simultaneously in real-time, it does not supply color (RGB) or thermal-IR information. Therefore, we propose a system that can generate 3D multi-spectrum data that include 3D shape, visible and thermal-IR information by registering three different kinds of cameras in real-time. The generated 3D multi-spectrum data created by the system are used for face recognition and to solve problems associated with variations in pose and light. With the proposed system, we can use four different kinds of information: (1) 3D depth data from the ToF camera; (2) near-infrared data from the ToF camera; (3) visible (RGB) data from the color camera and (4) thermal-IR data from the thermal-IR camera.

**Figure 2.**
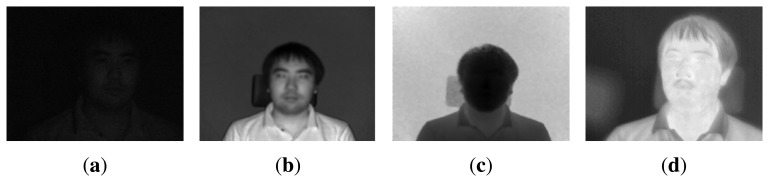
Captured images in a lightless condition; (**a**) visible range image; (**b**) near-infrared (IR) image from the time-of-flight (ToF) camera; (**c**) distance image from the ToF camera; and (**d**) thermal-IR image.

Since the thermal-IR and ToF cameras can capture thermal-IR (3–5 μm, 8–12 μm) and NIR(750 nm–1,400 nm) ranges regardless of the visible range (360 nm–820 nm), they can be used in extremely low light conditions. As shown in Figure 2, even though the image captured by the visible range camera is almost black in the dark, the images from the ToF and thermal-IR cameras are not affected by changes in external light. Therefore, there may be a wider range of uses for these cameras beyond the visible range, e.g., in surveillance applications, such as human detection and tracking at night. Even though IR and ToF cameras are almost invariant to light variation, they do not provide color or detailed texture information. Therefore, the proposed system has many advantages in terms of surveillance, robot vision and HCI, which require 3D information, as well as color and thermal-IR information in real-time.

### 3.2. Registration of ToF, Color and Thermal-IR Cameras

In this step, the registration is used to find visible and thermal-IR information corresponding to 3D data. Since the proposed system consists of three cameras located at different positions, each image from the sensors has different image coordinates. In addition, since each camera provides different features, such as color, thermal-IR, near-IR and 3D information, coordination among cameras is difficult.

**Figure 3.**
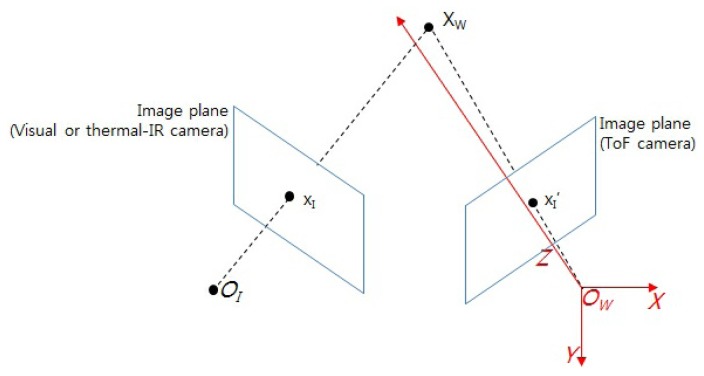
Relationship between a different coordinate system (world(*X_W_*) and image (*x_I_*, $x_{I}^{\prime }$) coordinate system).

Three-dimensional geometry, in which a 3D point (*X_W_*) in a world coordinate system is projected onto a 2D point (*x_I_*) in an image coordinate system, is used for registration between visible or thermal-IR and ToF cameras. As shown in Figure 3, since the origin of the world coordinate (*O_W_*) is the same as the origin of the ToF camera, a point in the world coordinate system can be represented as the camera coordinate system of the ToF camera. In other words, a world coordinate of a point in 3D space is directly acquired as the 3D point of the ToF camera, as described in Equation (1). Additionally, *x_I_* represents a 2D coordinate of the image (pixel) coordinate system of the visible camera. $x_{I}^{'}$ is a 2D coordinate of the image coordinate system of the ToF camera.

(1) $$X_{\mathrm{ToF}}=X_{W}={\left[\begin{array}{ccc} x & y & z \end{array}\right]}^{T}$$

(2) $$x_{I}={\left[\begin{array}{cc} u & v \end{array}\right]}^{T}$$

(3) $${x_{I}}^{\prime }={\left[\begin{array}{cc} u^{\prime } & v^{\prime } \end{array}\right]}^{T}$$

**Figure 4.**
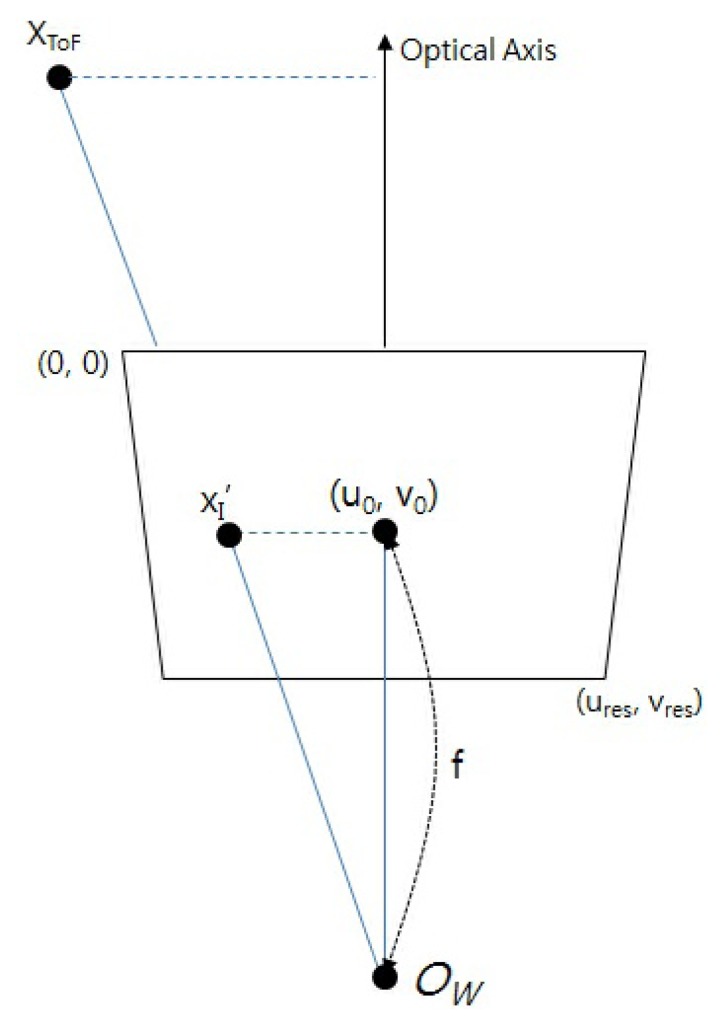
Structure of ToF camera: a 3D point (*X_ToF_*) is projected on a 2D point ( $x_{I}^{\prime }$) in the image plane of a ToF camera.

First, we estimate the world coordinate of a 3D point (*X_ToF_* ) from the image coordinate of a 2D point ( $x_{I}^{\prime }$) in the image plane of the ToF camera, as shown in Figure 4. In Figure 4, (*u*_0_,*v*_0_) is the principal point of the image plane and (*u_res_*, *v_res_*) represents the resolution of the distance image.

A 3D point (*X_ToF_*) is projected onto a 2D point ( $x_{I}^{\prime }$) on the image plane. Since the pixel value of a distance image from the ToF camera represents the distance between the camera and an object, we can obtain the distance information (*z*) from the images. Therefore, we can estimate the *x*-coordinate of the 3D point (*X_ToF_*) according to Equations (4) and (5). *f* is the focal length of the ToF camera, which can be calculated from the camera calibration [47].

(4) $$\frac{f}{z}=\frac{\left(u^{\prime }-u_{0}\right)}{x}$$

(5) $$x=\frac{(u^{\prime }-u_{0})\times z}{f}$$

The coordinate of the 3D point (*X_ToF_*) can be estimated using the same method as in Equation (6).

(6) $$y=\frac{(v_{0}-v^{\prime })\times z}{f}$$

Therefore, the world coordinate of the 3D point (*X_ToF_*) based on the distance image can be calculated using Equation (7).

(7) $$X_{\mathrm{ToF}}={\left[\begin{array}{ccc} x & y & z \end{array}\right]}^{T}={\left[\begin{array}{ccc} \frac{(u^{\prime }-u_{0})\times z}{f} & \frac{(v_{0}-v^{\prime })\times z}{f} & z \end{array}\right]}^{T}$$

The correspondence between *X_ToF_* and *x_I_* can be represented by Equation (8). *C* and *R* indicate the translation and rotation of the camera coordinate with respect to the world coordinate system, respectively. *C* is a 3 × 1 vector, and *R* is a 3 × 3 matrix. *K* is a 3 × 3 camera calibration matrix that includes the internal parameters of the camera [15].

(8) $$x_{I}=KR^{T}(X_{\mathrm{ToF}}-C)$$

Equation (8) can be represented as in Equation (9), which is a linear equation with a homogeneous coordinate.

(9) $$x_{I}=(KR^{T}|-KR^{T}C)(\begin{array}{c} X_{\text{ToF}}\\ 1 \end{array})$$

In summary, the relationship between *X_ToF_* and *x_I_* is given by Equation (10). The 3 × 4 matrix *P* = (*KT^T^*∣ – *KR^T^C*) is the projection matrix of the camera.

(10) $${\tilde{x}}_{I}=P{\tilde{X}}_{\text{ToF}}$$

where *X̃_ToF_* = [*x y z* 1]*^T^* is the homogeneous coordinate of the 3D point (*X_ToF_* = [ *x y z* ]*^T^*), which is a 4 × 1 column vector with one added as the last element of the vector, and *x̃_I_* = [ *u v* 1 ]*^T^* is the homogeneous coordinate of the 2D point (*x_I_* = [ *u v*]*^T^*), which is a vector.

**Figure 5.**
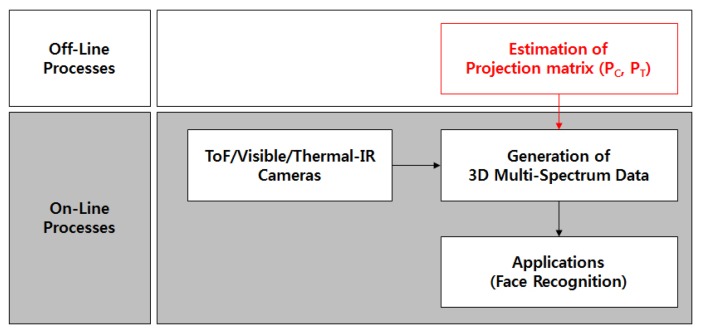
The proposed system can be divided into off-line and on-line processes.

We divide the proposed system into on-line and off-line steps, as shown in Figure 5. Before the on-line process, the projection matrices, which represent the relationship between the 3D coordinates of the ToF camera and the 2D projective coordinate of the visible (*P_C_*) and thermal-IR (*P_T_*) cameras, should be estimated off-line. Subsequently, 3D multi-spectrum data are generated and applications, such as face recognition, are completed on-line.

**Figure 6.**
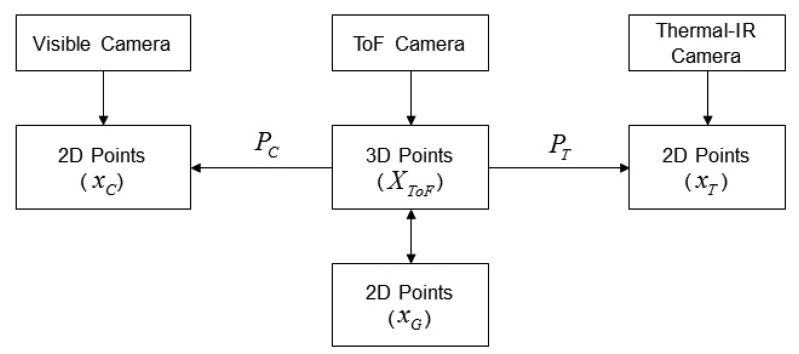
Registration between cameras.

In the proposed system, 3D points can be acquired from the ToF camera, and two kinds of 2D points can be obtained from the color and thermal-IR cameras. Therefore, the two camera projection matrices can be correctly estimated. One is a matrix (*P_C_*) representing the relationship between the 3D points and 2D points of the color images, as in Equation (11), and the other is a matrix (*P_T_*) based on the 2D points of the thermal-IR images, as in Equation (12).

(11) $${\tilde{x}}_{C}=P_{C}{\tilde{X}}_{\text{ToF}}$$

(12) $${\tilde{x}}_{T}=P_{T}{\tilde{X}}_{\text{ToF}}$$

where *x̃_C_* and *x̃_T_* are homogeneous coordinate representations of 2D points in the color image and the thermal-IR image, respectively, and *X̃_ToF_* is a homogeneous coordinate representation of a 3D point from the ToF camera. Figure 6 illustrates the main concept of the registration between cameras.

**Figure 7.**
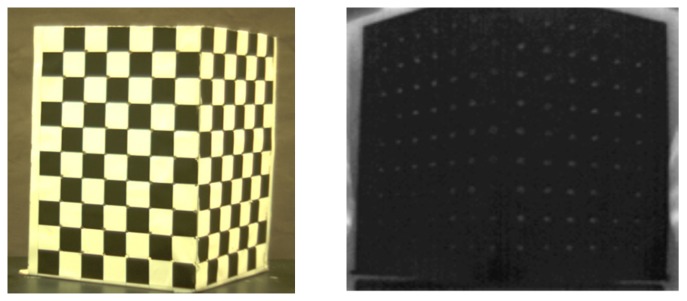
Images of the calibration rig taken by the visible camera (**left**) and the thermal-IR camera (**right**).

To estimate each projection matrix in an off-line process, the corresponding points having the same feature point in all three cameras should be extracted. To identify the corresponding points between different cameras, we use a calibration rig to extract feature points at the corners of a check pattern in a color image. We also make holes in the corners of the check pattern as feature points for the thermal-IR image. The 2D points (*x_C_*, *x_T_*) for color and thermal-IR can be extracted from each image of the calibration rig shown in Figure 7.

**Figure 8.**
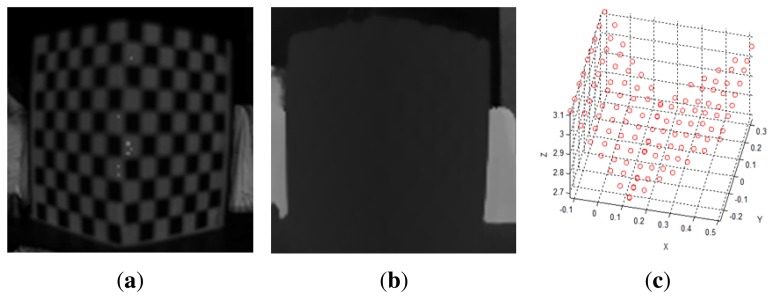
3D points from the ToF camera: (**a**) near-IR image of the calibration rig from the ToF camera; (**b**) distance image of the calibration rig from the ToF camera; (**c**) extracted 3D coordinates of the calibration rig at the corner point of the check pattern in the near-IR image.

Using a ToF camera, 3D points (*X_ToF_*) can be acquired directly, since the distance image has already been calibrated using the 2D points (*x_G_*) of the near-IR image from the ToF camera (Figure 8). Therefore, we first extract the feature points that are also the corner points of the check pattern in the near-IR image, as shown in Figure 8a. By using the points extracted from the near-IR images, the 3D coordinates can be extracted from the distance image, as in Figure 8b, as well as from the ToF camera, as shown in Figure 8c.

If we know the number of correspondences *x_i_* ↔ *X_i_*, *i* = 1, 2, … *N* (*N* ≥ 6) between the 3D points (*X_i_*) and the 2D image points (*x_i_*), we can estimate the camera projection matrix using the direct linear transformation (DLT) algorithm, which is a minimization method used to find an approximate linear solution by singular value decomposition (SVD) [47]. Since we can extract a number of correspondences using a calibration rig, we can estimate the camera projection matrices (*P_C_*, *P_T_*). If we know the projection matrix, then we can obtain each corresponding projected 2D image point on the visible and thermal-IR image planes from the 3D coordinates obtained with the ToF camera using Equations (2) and (3), thereby allowing for the acquisition of color and thermal-IR information corresponding to the 3D points.

### 3.3. Generation of 3D Multi-Spectrum Face Data

Before 3D multi-spectrum face data can be generated, the noise in 3D distance images from ToF camera must be addressed. We first perform a median filter to remove the salt and pepper type noise in every image. After that, we use an average image of 10 distance images to capture a more precise distance image.

We then apply the previous registration method to find the corresponding color and thermal-IR information to obtain the 3D points of the face to be recognized. In other words, we find the corresponding 2D coordinates of the color and thermal-IR images of the 3D points from the estimated projection matrix (*P_C_*, *P_T_*) in Equations (11) and (12). Finally, we generate 3D multi-spectrum face data, which include visible and thermal-IR textures with 3D shape information.

**Figure 9.**
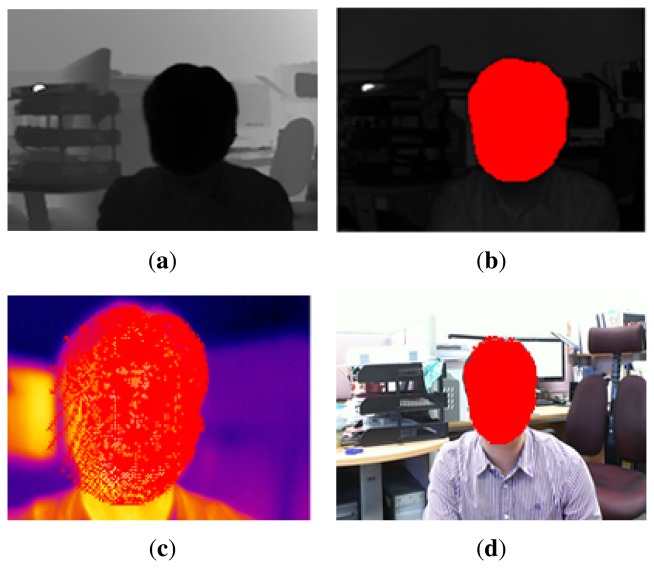
(**a**) 3D depth image and corresponding points in the near-IR image (**b**); thermal-IR image (**c**); and visible (color) image (**d**).

Figure 9 shows the corresponding distance image (3D shape information) (a) and near-IR image (b) of the thermal-IR image (c) and visible image (d). The red-cross points in Figure 9c,d represent projected correspondence points on the thermal-IR and visible images from the 3D face region in (b).

**Figure 10.**
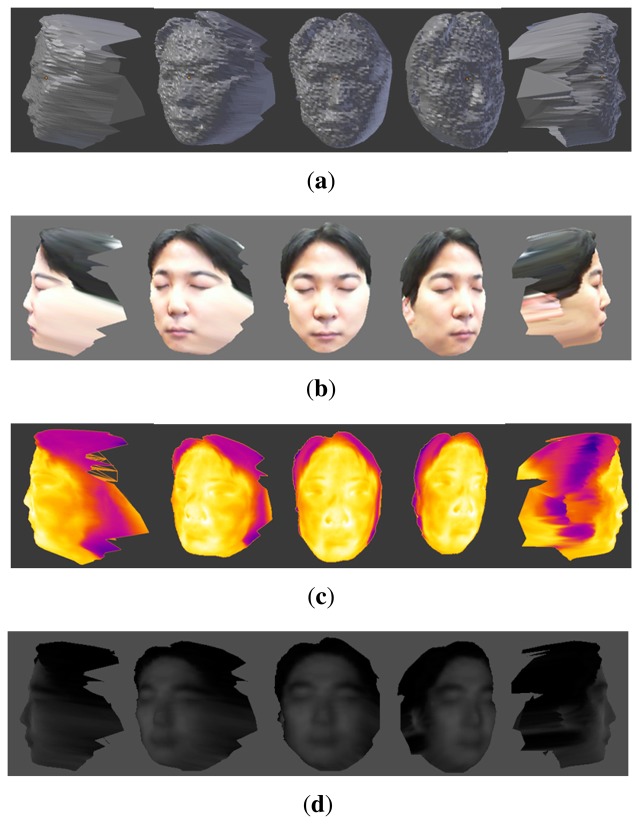
3D multi-spectrum face data: (**a**) 3D face shape data from the ToF camera; (**b**) color 3D multi-spectrum data; (**c**) thermal-IR 3D multi-spectrum data; and (**d**) near-IR 3D multi-spectrum data.

As a result of the registration, 3D multi-spectrum face data for face recognition can be generated. Since the resolution of the depth image from the ToF camera is too small, we generate 3D multi-spectrum face data by 3D mesh rendering in OpenGL to create more detailed 3D face data. Figure 10a shows visible and thermal-IR images, as well as two kinds of 3D multi-spectrum data. One image is color textured (Figure 10b) and the other is thermal-IR textured (Figure 10c). Since the coordinate of the distance image corresponds perfectly with the coordinate of the near-IR image, 3D multi-spectrum face data, including near-IR texture information, can also be generated, as shown in Figure 10d.

### 3.4. Face Recognition

**Figure 11.**
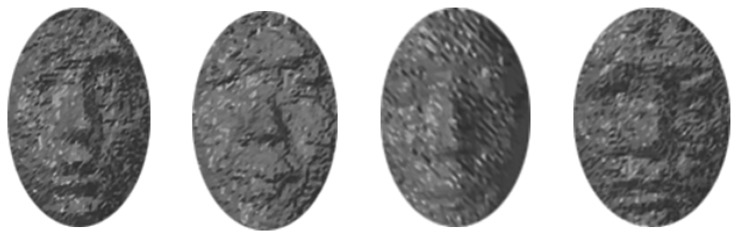
Samples of 3D face data acquired by the ToF camera.

Face recognition is performed using the generated 3D multi-spectrum face data. Then, 3D face data from the ToF camera contains certain distance noise, as shown in Figure 11. The performance of 3D face recognition is highly dependent upon distance noise [48]. Therefore, we perform a 2.5D face recognition step, which includes a 2D frontal face image projected from the transformed 3D multi-spectrum face data by a normalization step.

Before recognizing a face, a normalization step to transform rotated faces into reference posed faces is necessary to reduce the pose variation. There are many pose estimation algorithms for minimizing the mean square error between points in reference data and the closest points in input data by using translation, rotation and scaling [49]. We use an iterative closest points (ICP) algorithm, which is one of the most commonly used algorithms for registering 3D data [50,51]. In order to evaluate 2.5D face recognition, we apply four kinds of classification methods (listed below) that are commonly used for 2D face recognition.

1. Principal component analysis (PCA)
2. Fisher linear discriminant analysis (FLDA)
3. PCA feature extraction + support vector machine (PCA + SVM)
4. PCA feature extraction + reduced multivariate polynomial pattern classifier (PCA + RM) [52].

## 4. Experiments

In order to evaluate the proposed 3D multi-spectrum face-data-based recognition system, we compare the performance of our proposed system with several existing face recognition methods using different face data, including 2D/3D and color/thermal-IR face data. We separate the experiments into pose and light variations in order to ensure the robustness of our proposed approach.

### 4.1. Experimental Environments

**Figure 12.**
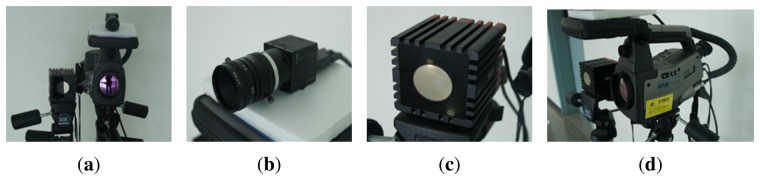
The proposed system: (**a**) proposed full system; (**b**) visible camera; (**c**) ToF camera; and (**d**) thermal-IR camera.

In order to obtain 3D information and near-IR images in real-time, we use a SR-4000 ToF camera with MESAimaging that can provide a resolution of 176 × 144 pixels at 30 FPS. Color images of the scene are captured using a FL2 by Point Grey that provides a resolution of 1, 024×768 pixels at 30 FPS. Thermal-IR images are acquired from a ThermaCAM S65 with an FLIRsystem that has a resolution of 320 × 240 pixels at 50/60 Hz. Since each sensor operates with a different frame rate, we need to set temporal synchronization among the sensors. We adjust the timing for the image capturing of visual and thermal-IR cameras to the timing of the image capturing of the ToF camera. The SR-4000 ToF camera supports a software trigger mode that uses the callback mechanism to capture depth images. When the callback function is called upon, all images are captured by multi-thread. Although we had tried to set the temporal correspondence by multi-thread, 2D image capturing and depth image capturing are, respectively, started with a small time difference, which is about 1*msec*. Figure 12 shows the installation of the three cameras. The ToF camera in Figure 12c is positioned to the right of the thermal-IR and visible cameras. The visible camera in Figure 12b is set next to the thermal-IR camera, as shown in Figure 12d. Each camera is located as close as possible to reduce occlusion caused by the different camera positions.

**Figure 13.**
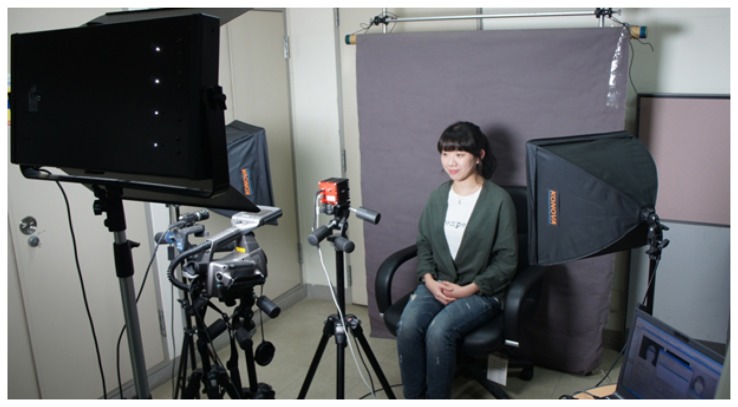
Experimental environments: three light sources and three different kinds of cameras.

Figure 13 shows our experimental environments. The distance between the face image to be acquired and the cameras is set at 1 m. Three light sources are used to create light variation. In addition, a dark screen is installed in the background to make it easy to separate face images from the background.

**Figure 14.**
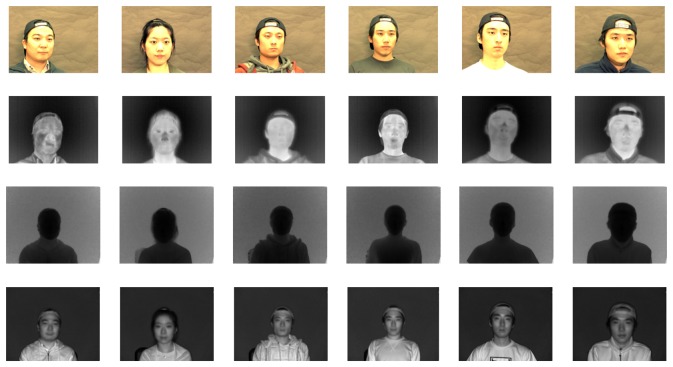
Example images in our face database including 3D, visible and thermal-IR information. The first row shows some color images from the visible camera. The second row shows images from the thermal-IR camera. Distance images from the ToF camera are shown in the last row.

Even though the data acquisition system is implemented in C, OpenGLand OpenCV, which is a real-time processing library for computer vision by Intel, other steps, including normalization and recognition, were simulated using Matlab 2009b on a machine with a 2.93 GHz Intel Core i7 870 and 4 GB of physical memory. Since there is no public facing database that includes all registered 3D, visible and thermal-IR information with variable illumination and pose conditions, we created two databases with five different poses and five levels of light variation. All images for the database are captured indoor with daylight conditions. Each database contains 500 3D multi-spectrum face datasets obtained from 100 subjects (5 (variations) × 100 (subjects) = 500). We detect face region using the Viola-Jones face detector, which was implemented using OpenCV [53]. All face images are normalized to 50 × 50 pixels for recognition. Figure 14 shows sample images from our database, which consists of distance, visible and thermal-IR images. The first row in Figure 14 shows color (RGB) images from the FL2 camera. The second row shows the thermal-IR images captured by the ThermaCAM S65. The third and fourth rows show the distance and near-IR images, respectively, from the SR-4000 ToF camera.

### 4.2. Estimation of Projection Matrices of the Visible and Thermal-IR Cameras

**Figure 15.**
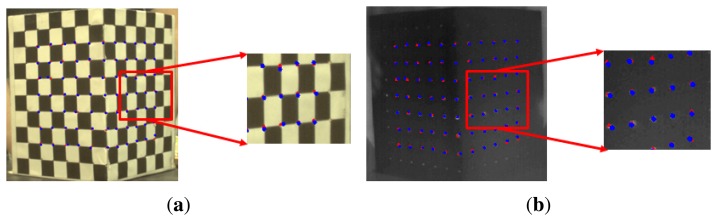
Re-projection error between projected 2D coordinates from 3D points and 2D points extracted from the (**a**) visual image and (**b**) thermal-IR image. The red and blue points represent extracted 2D points and projected 2D points, respectively.

An average image derived from the 100 ToF camera distance images is used to estimate precise projection matrices after applying a median filter to reduce the effects of distance noise. Once the projection matrices have been estimated in the off-line process, it does not need to be operated again. To estimate the projection matrix, we extract 77 corresponding points from the visible, thermal-IR and distance images. After that, we estimate the projection matrices of the visible and thermal-IR images with respect to the 3D points from the ToF camera. The accuracy of the projection matrices is evaluated as re-projection error, which is the Euclidean distance between the projected 2D coordinates from Equations (11) and (12) and the 2D coordinates of extracted points in the visible and thermal-IR images, as shown in Figure 15.

We measured re-projection errors at different visual and thermal-IR camera positions, but with the ToF camera fixed. The mean values of 30 repetitions of re-projection errors with the visual and thermal-IR camera are 2.7308 pixels and 1.5629 pixels, respectively. Since the resolution of the visual image is larger than that of the thermal-IR image, the re-projection error of the visual image is more sensitive to noise than the thermal-IR image. There are many reasons why re-projection error is generated. First, the low resolution (176 × 144) of the ToF camera causes more error in high-resolution images during the feature extraction step. Second, even though an average of 30 distance images is used to extract 3D points, the values still might contain distance noise. Therefore, distance noise estimation and precise feature extraction algorithms are needed to estimate precise projection matrices.

### 4.3. Face Recognition with Pose Variation

**Figure 16.**
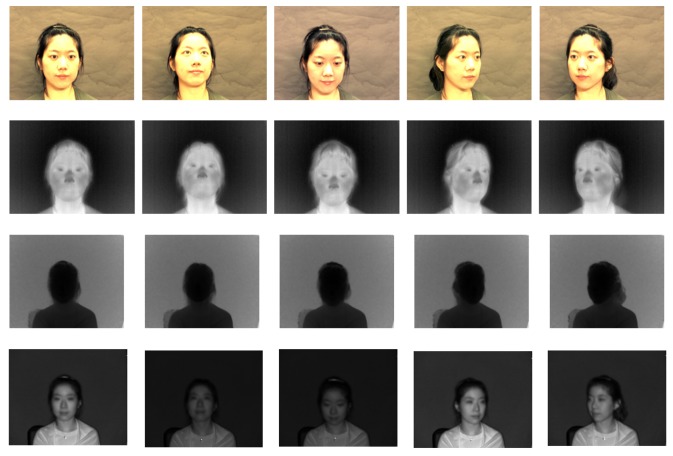
Example images from the database of five different facial poses: front, up, down, right and left.

In this experiment, we only consider pose variation in a face regardless of illumination change. Therefore, only pose differences in the face database are used. Lighting conditions remain constant. To verify the robustness against pose variation, we generated a database consisting of 100 subjects, each exhibiting five different poses (left, right, up, down and front) with respect to the ToF camera for a total of 500 images. Figure 16 shows example images of a few subjects in various poses.

**Figure 17.**
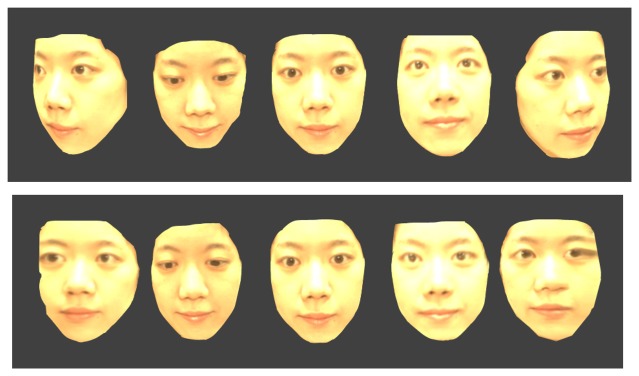
The first row shows 3D visible multi-spectrum face data before normalization and the second row shows normalized 3D visible multi-spectrum face data.

**Figure 18.**
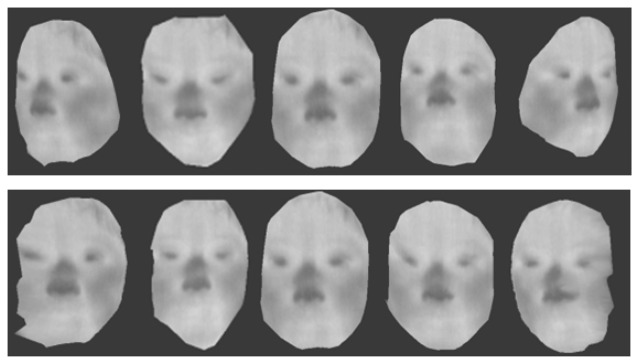
The first row shows 3D thermal-IR multi-spectrum face data before normalization, and the second row shows normalized 3D thermal-IR multi-spectrum face data.

**Figure 19.**
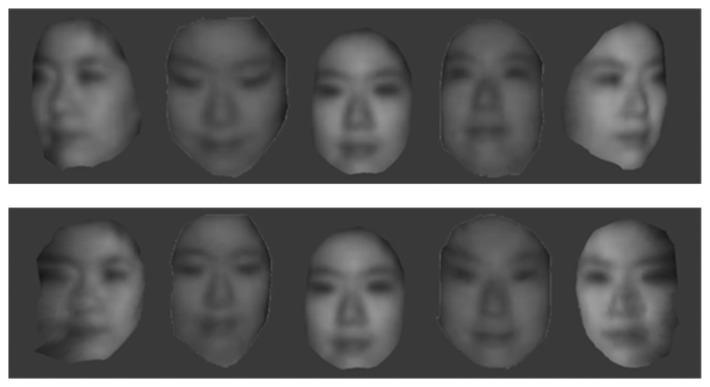
The first row shows 3D near-IR multi-spectrum face data before normalization, and the second row shows normalized 3D near-IR multi-spectrum face data.

Using those images, we can generate 3D multi-spectrum face data and perform normalization by ICP. Normalized face data are shown in Figures 17, 18 and 19, which were constructed using visible, thermal-IR and near-IR multi-spectrum data, respectively.

Experiments using five-fold cross validation are performed to verify the face images. That is, four face images of a person are used for training, and then, one face image is used for testing. We first train each classifier using 400 face data images, with four posed data images per subject, and perform the test using 100 other face data images in different poses. Recognition is performed using a nearest neighbor classifier with PCA, FDA, PCA + SVM and PCA + RM.

To compare the proposed 2.5D face recognition using 3D multi-spectrum face data with other recognition methods, we calculate the recognition rate for different face data by PCA, FDA, PCA + SVM and PCA + RM. In this experiment, we have used different polynomial orders 1 (RM_1_), 2 (RM_2_) and 3 (RM_3_). Since order 3 shows saturated performance, the experiment stops at order 3. In the SVM and RM experiments, we extracted features using PCA and adopt SVM and RM as classifiers. In the SVM experiments, we adopt a linear model, a polynomial model and a radial basis function as kernels. The parameters, such as the number of principal components in the eigenface and the number of support vectors with SVM, are experimentally selected to achieve the lowest error rate with each method. Five types of experiments are performed to observe the performance and the robustness against pose variation using 3D multi-spectrum face data.

1. 2D visible face images with pose variation (2D-vis)
2. 3D range images with pose variation (3D)
3. 3D range images after ICP normalization (3D + ICP)
4. Projected 2D visible face images from 3D multi-spectrum data after ICP normalization (2D-vis + ICP)
5. Projected 2D thermal-IR face images from 3D multi-spectrum data after ICP normalization (2D-the + 3D + ICP)
6. Projected 2D near-IR face images from 3D multi-spectrum data after ICP normalization (2D-NIR + 3D + ICP)

The approach in (5) is our proposed method. All experiment results are shown in Table 1.

**Figure 20.**
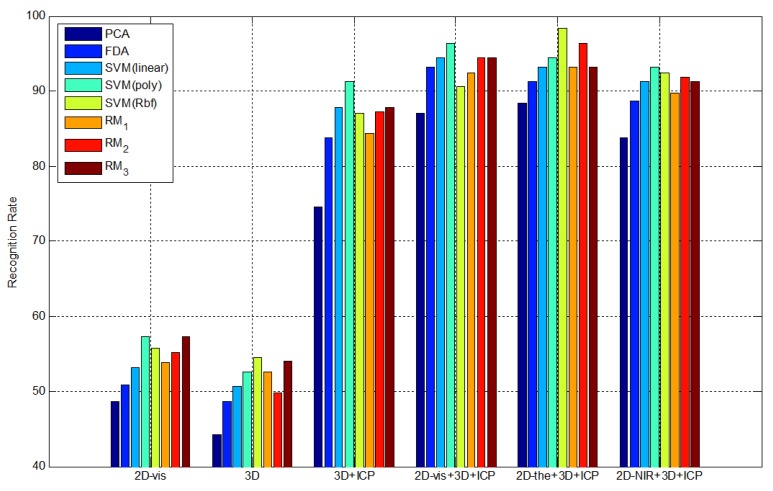
Recognition rate in terms of recognition approaches.

Based on these experiments, we can solve the pose variation problem by normalizing the 3D face data. Since there is no light variation to evaluate in this experiment, the result of using 3D visible face data (4), and 3D thermal-IR face data (5) yield similar recognition rates as the highest recognition rate, as shown in Figure 20. Even though the result of (3) uses 3D information, the recognition rate is not as high, because the range images from the ToF camera contain more distance noise. Among the classifiers, the SVM and RM classifiers show the best recognition rate.

### 4.4. Face Recognition with Light Variation

**Figure 21.**
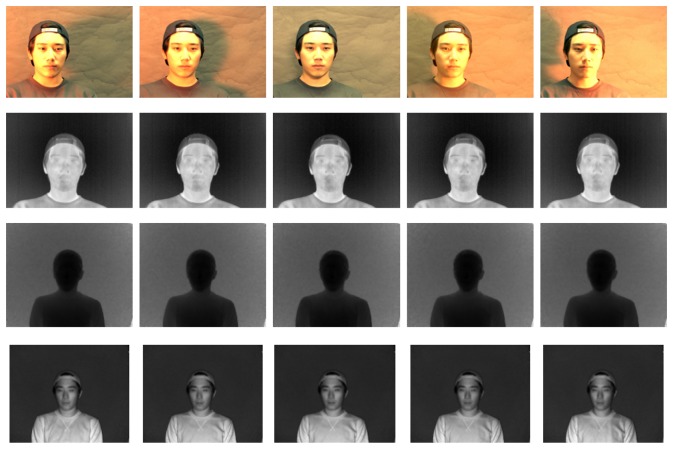
An example from the face database having five different light angles: 90° left, 45° left, front, 90° right and 45° right.

We verify performance with light variation using a database consisting of images of 100 subjects having five different levels of light variation (30° left, 15° left, front, 30° right and 15° right) without pose variations, as shown in Figure 14. This experiment does not require a normalization step for 3D multi-spectrum face data. Figure 21 shows the invariance of the thermal-IR images with illumination change, which strongly influences the visible image.

The experiments are performed using five-fold cross-validation, meaning that one face image is used for testing, and the other four face images are used for training to verify the face image. Therefore, 400 images were used for training, and 100 were used for testing. The experimental methods are the same as for face recognition with pose variation.

To compare the proposed 2.5D face recognition using 3D multi-spectrum face data with other methods, we calculate the recognition rate of each method by PCA, FDA, PCA + SVM and PCA + RM. Five types of experiments are performed to show the robustness of the proposed method against light variation using 3D multi-spectrum face data as follows:

1. 2D visible face images with light variation (2D-vis)
2. 2D thermal-IR face images with light variation (2D-the)
3. 3D range images with light variation (3D)
4. Projected 2D visible face images from 3D multi-spectrum data with light variation (2D-vis + 3D)
5. Projected 2D thermal-IR face images from 3D multi-spectrum data with light variation (2D-the + 3D)
6. Projected 2D near-IR face images from 3D multi-spectrum data with light variation (2D-NIR + 3D)

**Figure 22.**
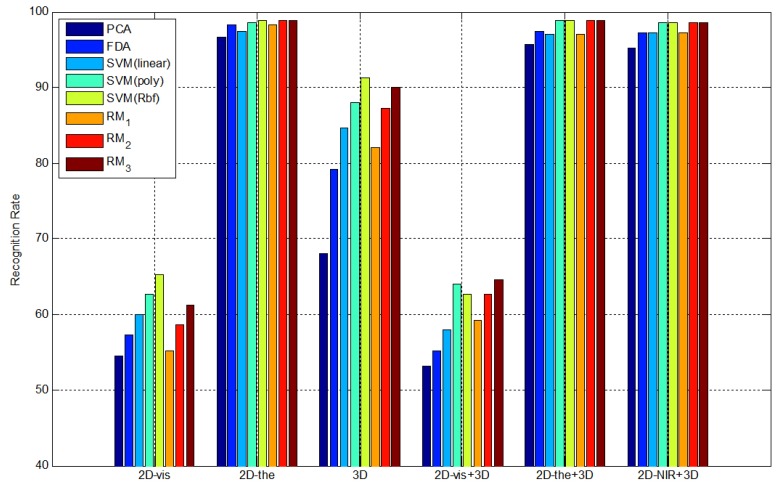
Recognition rate in terms of recognition approaches.

All recognition rates are shown in Table 2. The results of the experiments indicate that thermal-IR texture is invariant to light variation, even though visible texture is strongly affected by surrounding light conditions. In addition, the range image, which is not changed by illumination, shows a reliable recognition rate, but not as high as the recognition rate of thermal-IR images, as the range data includes distance noise. As shown in Figure 22, the result using the proposed method (5) and the 2D thermal face image (2) shows the robustness against light variation.

Based on these experiments, the proposed 2.5D face recognition technique with 3D multi-spectrum face data shows better performance than 2D and 3D face recognition with variation in pose and light. The proposed face recognition approach improves the recognition rate with light variation, since the thermal-IR pattern of the face is not changed by visible illumination changes. Even though normalized frontal face data can be generated from 3D information, the occluded region between the cameras and the face cannot be reconstructed. Therefore, some distortions in normalized face images can be generated by normalization of the occluded region. This may cause a slight performance reduction with pose variation. This problem can be solved by using additional ToF cameras at different locations to cover the occluded face regions.

### 4.5. Processing Time

The processing time is an important factor for a real-time sensor system. The processing time for each step is shown in Table 3. Each processing time is calculated by averaging of 100 attempts.

The whole system can be divided into two subsystems: a 3D multi-spectrum sensor system and a face recognition system. The image acquisition and 3D multi-spectrum data generation are implemented by C language, and the recognition part is implemented by Matlab. The 3D multi-spectrum sensor system takes 0.07 s (about 15 data points per second) to generate a 3D multi-spectrum data. In recognition, the processing time is less than 1 msec. Therefore, the proposed 3D multi-spectrum sensor system can be used with various real-time applications, such as robots and surveillance.

## 5. Conclusions

In this paper, we propose a novel 3D multi-spectrum sensor system that provides registered visible, near-IR, thermal-IR and 3D information in real time. By using this information, we can design more flexible and robust systems in terms of selecting sensor combinations and more effective fused features. We showed the usefulness of the proposed system for a face recognition system design with variations in pose and illumination. This system may also be very useful for designing vision systems for surveillance, 3D object modeling and object recognition.

## Figures and Tables

**Table 1.** Recognition rate with respect to pose variation. ICP, iterative closest points; PCA, principal component analysis; FLDA, Fisher linear discriminant analysis; SVM, support vector machine.

**Case**	**2D-vis**	**3D**	**3D + ICP**	**2D-vis + 3D + ICP**	**2D-the + 3D + ICP**	**2D-NIR + 3D + ICP**
PCA	48.6% (243/500)	44.2% (221/500)	74.6% (373/500)	87% (435/500)	**88.4**% (442/500)	83.8% (419/500)

FLDA	50.8% (254/500)	48.6% (243/500)	83.8% (419/500)	**93.2**% (466/500)	91.2% (456/500)	88.6% (443/500)

PCA + SVM (linear)	53.2% (266/500)	50.6% (253/500)	87.8% (439/500)	**94.4**% (472/500)	93.2% (466/500)	91.2% (456/500)
PCA + SVM (poly)	57.2% (286/500)	52.6% (263/500)	91.2% (456/500)	**96.4**% (482/500)	94.4% (472/500)	93.2% (466/500)
PCA + SVM (Rbf)	55.8% (279/500)	54.6% (273/500)	87% (435/500)	90.6% (453/500)	**98.4**% (492/500)	92.4% (462/500)

PCA+RM_1_	53.8% (269/500)	52.6% (263/500)	84.4% (422/500)	92.4% (462/500)	**93.2**% (466/500)	89.8% (449/500)
PCA+RM_2_	55.2% (276/500)	49.8% (249/500)	87.2% (436/500)	94.4% (472/500)	**96.4**% (482/500)	91.8% (459/500)
PCA+RM_3_	57.2% (276/500)	54% (270/500)	87.8% (439/500)	**94.4**% (472/500)	93.2% (466/500)	91.2% (456/500)

**Table 2.** Recognition rate with respect to light variation.

**Case**	**2D-vis**	**2D-ther**	**3D**	**2D-vis + 3D**	**2D-ther + 3D**	**2D-NIR + 3D**
PCA	54.6% (273/500)	**96.6**% (483/500)	68.0% (340/500)	53.2% (266/500)	95.6% (478/500)	95.2% (476/500)

FLDA	57.2% (286/500)	**98.2**% (491/500)	79.2% (396/500)	55.2% (276/500)	97.4% (487/500)	97.2% (486/500)

PCA + SVM (linear)	60.0% (300/500)	**97.4**% (491/500)	84.6% (423/500)	58.0% (290/500)	97.0% (485/500)	97.2% (486/500)
PCA + SVM (poly)	62.6% (313/500)	98.6% (493/500)	88.0% (440/500)	64.0% (320/500)	**98.8**% (494/500)	98.6% (493/500)
PCA + SVM (Rbf)	65.2% (326/500)	**98.8**% (494/500)	91.2% (456/500)	62.6% (313/500)	**98.8**% (494/500)	98.6% (493/500)

PCA + RM_1_	55.2% (276/500)	**98.2**% (491/500)	82.0% (410/500)	59.2% (296/500)	97.0% (485/500)	97.2% (486/500)
PCA + RM_2_	58.6% (293/500)	**98.8**% (494/500)	87.2% (436/500)	62.6% (313/500)	**98.8**% (494/500)	98.6% (493/500)
PCA + RM_3_	61.2% (306/500)	**98.8**% (494/500)	90.0% (450/500)	64.6% (323/500)	**98.8**% (494/500)	98.6% (493/500)

**Table 3.** Processing time for each process.

**Content**		**Processing Time (*msec*)**
Image acquisition		29
3D multi-spectrum data generation		41
Recognition	PCA	0.31
	FLDA	0.38
	PCA + SVM (linear)	0.62
	PCA + SVM (poly)	0.75
	PCA + SVM (rbf)	0.89
	PCA + RM_1_	0.55
	PCA + RM_2_	0.63
	PCA + RM_3_	0.74
